# Disease burden and related medical costs of rotavirus infections in Taiwan

**DOI:** 10.1186/1471-2334-6-176

**Published:** 2006-12-15

**Authors:** Chun-Yi Lu, Tsai-Ling Lauderdale, Yin-Hua Fang, Chung-Yi Wang, Yu-Huai Ho, Che-Lun Hung, Luan-Yin Chang, Chin-Yun Lee, Li-Min Huang

**Affiliations:** 1 Department of Pediatrics, National Taiwan University Hospital, Taipei, Taiwan; 2 Division of Clinical Research, National Health Research Institutes, Zhunan, Taiwan; 3 Department of Pediatrics, Min-Shen Hospital, Tao-Yun, Taiwan; 4 Department of Pediatrics, Buddhist Tzu-Chi General Hospital, Hua-Lien, Taiwan

## Abstract

**Background:**

The disease burden and associated medical costs of rotavirus infections in inpatient and outpatient sectors in Taiwan were examined in anticipation of the availability of new rotavirus vaccines.

**Methods:**

The yearly national case number and medical costs for all for inpatients and outpatients with acute gastroenteritis (AGE) were extracted from the Bureau of National Health Insurance database in Taiwan according to ICD-9-CM codes. A retrospective study was also performed using records of children with AGE seen at three hospitals in Taiwan in 2001 to identify laboratory confirmed rotavirus infection cases. The annual incidence and related medical costs of AGE due to rotavirus infection were then estimated.

**Results:**

Children <5 years old comprised 83.6% of inpatient and 62.0% of outpatient pediatric AGE cases in Taiwan in 2001. Rotavirus was the most common agent detected among AGE patients in this age group in the three hospitals, and was detected in 32.9% (221/672) of inpatient and 24% (23/96) of outpatient stool specimens tested for microbial etiologies. An estimated 277,400 to 624,892 cases of rotavirus infections sought medical care in Taiwan in 2001, equaling one in 2 to 5 children <5 years old required medical care due to rotavirus infection. The incidence of hospitalization due to rotavirus infections was 1,528–1,997/100,000 for children <5 years old. The total associated medical costs due to rotavirus infection were estimated at US $10–16 millions in Taiwan in 2001. Although the per-capita medical cost of rotavirus infection was lower in Taiwan than in the United States or Hong Kong, the personal economic burden was similar among the three places when normalized for gross national incomes per capita.

**Conclusion:**

Infections caused by rotavirus constitute an important human and economic burden among young children in Taiwan. A safe and effective vaccine is urgently needed.

## Background

Rotavirus is the leading cause of severe childhood diarrhoea in developed and developing countries worldwide. Virtually all children could be infected before their fifth birthdays. It is estimated that each year rotavirus infections are responsible for approximately 2 million hospitalizations in children <5 years old worldwide [1,2]. The World Health Organization estimated that in 2002, over 400,000 deaths due to rotavirus in children <5 years old were preventable by vaccination [3]. Thus universal childhood immunization with rotavirus vaccine is expected to substantially reduce the mortality and morbidity due to rotavirus infections in immunocompetent children [4,5].

Rotavirus infections have been reported to occur year-round in Taiwan [6,7]. Recently, in the first report from the Asian Rotavirus Surveillance Network (ARSN) by Bresee et al, rotavirus infection accounted for 28% to 59% (mean 45%) of children <5 years old admitted to hospitals due to acute diarrhoea in Asia [8]. In a concurrent study of ARSN, rotavirus was detected in 43% of stool samples tested for etiology in children <5 years old admitted to 4 sentinel hospitals in Taiwan [9]. However, the national incidence of rotavirus disease in Taiwan is unknown, especially in the outpatient sector.

Taiwan implemented a national health insurance program in 1995, and by December 2000, over 96 % of its >22-million population was covered under this universal healthcare system [10]. The Bureau of National Health Insurance (BNHI) in Taiwan maintains an extensive database on all inpatient and outpatient visits, including their medical diagnoses and medical expenditure. In anticipation of the availability of new rotavirus vaccines, we performed a retrospective study using available data from three hospitals and the BNHI database to estimate the burden of rotavirus disease and its associated medical costs to determine the potential economic impact with the implementation of a national vaccination program in Taiwan.

## Methods

### Hospital data

Three hospitals in Taiwan participated in this study. The National Taiwan University Hospital (NTUH) is a large 2,000-bed medical center located in the highly urbanized city of Taipei. The Min-Shen Hospital (MSH) is a regional hospital with 600 beds located in Tao-Yuan City. This is a satellite city of Taipei and includes more suburban areas. Both of these hospitals are located in the most densely populated northern regions of Taiwan. The Buddhist Tzu-Chi General Hospital (TCH) is a 900-bed hospital in Hua-Lien City, which is located in the mountainous area of eastern Taiwan and serves a large rural area. This study was done independently of the ARSN studies.

We extracted medical and administrative records of all pediatric patients <5 years old with the principle diagnosis of acute gastroenteritis (AGE) who were seen in the outpatient department (including emergency rooms), and those admitted to these three hospitals between March 2001 and February 2002. While estimating the percentage of AGE attributable to rotavirus infections, patients whose records appeared both in the outpatients and inpatient sectors were counted as a single case in the inpatient sector. Only those whose stool samples were tested for both rotavirus and bacterial agents were taken into account. The causative microbes for AGE from patients who had stool specimens tested for microbial etiology were analyzed.

### Laboratory studies

Stool samples submitted for microbial etiology testing were tested for rotaviral antigen by monoclonal antibody incorporated enzyme-linked immunosorbent assay according to the manufacturer's instructions (Rotaclone, Meridian Diagnostic, Cincinnati, OH, USA). Bacterial cultures were performed using MacConkey, xylose-lysine-desoxycholate, Hektoen enteric and thiosulfate-citrate-bile salts-sucrose agar, and *Campylobacter *Agar plates (BBL, Becton Dickinson Microbiology System, Cockeysville, MD, USA) following standard clinical microbiology protocols.

### Data from the Bureau of National Health Insurance (BNHI) database

All the identification numbers of individuals were scrambled at BNHI before the database was made available for research purposes through a contract with the National Health Research Institutes. Because the database inclusive of all patient visits is tremendously large, we used the sampling database to perform the queries. The sampling database is a systematic sampling of every 500th outpatient visit and every 20th inpatient visit extracted from the all-patient records. For 2001, there were over 620,000 outpatient and over 140,000 inpatient records in the sampling database.

The BNHI database contains registration files, original claim information and reimbursement data. Medical diagnoses are coded according to the International Classification of Diseases, 9th revision, Clinical Modification (ICD-9-CM). We queried for patients 16 years old and younger with the following ICD-9-CM diarrhoea-associated diagnoses codes to analyze the number of AGE cases. The reimbursements associated with each visit and admission for these patients were extracted and analyzed. The codes include: 001 (cholera), 002 (typhoid and paratyphoid fever), 003 (salmonellosis), 004 (shigellosis), 005 (other bacterial food poisoning), 006 (amoebiasis), 007 (other protozoan intestinal diseases), 008 (intestinal infections due to other organisms including 00861 enteritis due to rotavirus), 009 (ill-defined intestinal infections), 558.9 (other and unspecified noninfectious gastroenteritis and colitis), and 787.91 (diarrhoea). Cases with the following codes were excluded in the current study: 003.2 (localized salmonella infections), 006.2 (amoebic liver abscess), 006.3 (hepatic amoebiasis), 006.4 (amoebic lung abscess), 006.5 (amoebic abscess of lung and liver), and 006.6 (amoebic skin ulceration).

### Estimation of Rotavirus Disease Burden and Medical Costs

Because the majority of rotavirus infections occurs in children <5 years old, surveillance of rotavirus infection usually defines a case of rotavirus diarrhoea as watery diarrhoea in a child <5 years old whose stool sample tested positive for rotavirus [11,12]. We adopted this rule and only cases of AGE in children <5 years old were taken into account in estimating disease burden or medical cost of rotavirus infections. The total number of rotavirus AGE cases was estimated by multiplying the total number of AGE cases in <5 years old children from the BNHI database and the percentages of laboratory confirmed rotavirus infections. Results from this study as well as those from the literature were used for this purpose.

Medical care expenses are reimbursed by the BNHI in Taiwan. The co-payment data (10% co-payments for hospitalized patients and US $1.5–3.0 for outpatients) are listed in the BNHI database along with reimbursement data and were included in our medical cost estimation. Nationwide total medical costs attributable to rotavirus infections in 2001 were estimated by multiplying the average BNHI reimbursements per AGE patient with the estimated annual case numbers of rotavirus infections. The monetary values in New Taiwan dollars (NT $) were converted into United States dollars (US $) based on the average exchange rate in 2001 (1 US $ = 33.8 NT $). The study was approved by the Clinical Research Ethics Committee of the National Taiwan University Hospital.

## Results

### National Health Insurance System Data

The nationwide case numbers of acute gastroenteritis (AGE) derived from the BNHI database is shown in Table 1. There were a total of 2,829,740 pediatric patient visits due to AGE, including 2,750,500 (97.2%) in the ambulatory and 79,240 (2.8%) in the inpatient sector in Taiwan in 2001. The majority of AGE occurred in children <5 years old, with this age group accounting for 62.0% (1,704,000/2,750,500) and 83.6% (66,260/79,240) of pediatric outpatients and inpatients with AGE, respectively. However, most of the AGE cases were not coded for a specific etiologic agent. Instead, the most common codes reported were "Other and unspecified noninfectious gastroenteritis and colitis" (ICD-9-CM 558.9) and "Ill-defined intestinal infections" (ICD-9-CM 009). These two codes together accounted for 95.3% [(836,500+1,783,500)/2,750,500] of outpatients and 82.0% [(9,500+55,460)/79,240] of inpatients with AGE related diagnoses.

**Table 1:** Case numbers of acute gastroenteritis with various ICD-9-CM codes in children in Taiwan, 2001^a^.

Sectors	Age (years old)	Case numbers of various acute gastroenteritis associated ICD-9-CM^b^											%^d^
			
		001	002	003	004	005	007	008^c^	009	558.9	787.91	Total	
Outpatient	<5	500	1,000	40,000	500	1,000	500	34,500	540,000	1,085,500	500	1,704,000	62.0
	5–16	0	0	20,500	0	5,000	0	24,000	296,500	698,000	2,500	1,046,500	38.0
	0–16	500	1,000	60,500	500	6,000	500	58,500	836,500	1,783,500	3,000	2,750,500	
													
Inpatient	<5	0	20	4,320	100	40	0	8,180	8,220	45360	20	66,260	83.6
	5–16	0	0	440	200	80	0	880	1280	10100	0	12,980	16.4
	0–16	0	20	4,760	300	120	0	9060	9500	55460	20	79,240	
													
Total	<5	500	1,020	44,320	600	1040	500	42680	548220	1,130,860	520	1,770,260	62.6
	5–16	0	0	20,940	200	5080	0	24880	297,780	708100	2500	1,059,480	37.4
	0–16	500	1,020	65,260	800	6,120	500	67,560	846,000	1,838,960	3,020	2,829,740	

^a ^Extrapolated from the Bureau of National Health Insurance 2001 sampling database in Taiwan (see Methods for details).

^b^ ICD-9-CM: International Classification of Diseases, 9th revision, Clinical Modification. 001, cholera; 002, typhoid and paratyphoid fever; 003, salmonellosis [excluding 003.2 (localized *Salmonella *infections)]; 004, shigellosis; 005, other food poisoning (bacterial); 007, other protozoan intestinal diseases; 008, intestinal infections caused by other organisms; 009, ill-defined intestinal infections; 558.9, other and unspecified noninfectious gastroenteritis and colitis; 787.91, diarrhoea. There were no cases of 006 (amoebiasis) reported.

^c ^Including 320 cases of ICD-9-CM 008.61, rotavirus.

^d ^Percentage of all 0–16 years old cases.

Enteritis due to rotavirus (ICD-9-CM 00861) was not reported in any outpatients and was reported only in a small number (0.4%, 320/79,240) of inpatients. All of the rotavirus AGE inpatients were in children <5 years old. Therefore, the prevalence of rotavirus AGE could not be obtained directly from the BNHI database. Because the majority of pediatric AGE cases occurred in <5 years old children, and because enteritis due to rotavirus was reported only in this age group in the BNHI database, our subsequent analysis on rotavirus disease burden focused on this age group.

### Hospital data

During the one-year study period, 5,015 children <5 years old with AGE were treated at the three participating hospitals. Of these 5,015 patients, 24.3% (1,219) were admitted due to severe diarrhoea and/or vomiting with dehydration, and were hospitalized for 3 to 5 days (mean = 4 days). Although laboratory diagnosis for etiology was performed on only a small proportion of the outpatients (2.5%, 96/3,796), over half of the hospitalized patients (55.1%, 672/1,219) were tested for an etiologic agent including both rotavirus and bacteria (Table 2). In AGE inpatients, rotavirus accounted for >33% in the 2 hospitals in the north. Although it was detected in lower proportion (18.3%) in the hospital in the east (TCH), it was still the most common agent detected in that hospital. Overall, rotavirus was the leading cause of AGE in these three hospitals, accounting for 24.0% (23/96) of outpatients and 32.9% (221/672) of inpatients tested.

**Table 2:** Number of cases and distribution of etiological diagnoses in children <5 years old with acute gastroenteritis (AGE) seen at three hospitals in Taiwan in 2001^a^.

Cases of AGE in:	NTUH	MSH	TCH	Total of 3 hospitals
Outpatients	1,274	1,679	843	3,796
Tested for etiology, N^b^	67	12	17	96
Positive for rotavirus, N (%)^c^	18 (26.9)	2 (16.7)	3 (17.6)	23 (24.0)
Positive for *Salmonella*, N (%)	9 (13.4)	2 (16.7)	4 (23.5)	15 (15.6)
Positive for *Shigella*, N (%)	0	0	0	0
Positive for *Campylobacter*, N (%)	1 (1.5)	NT^d^	NT	-
Inpatients	408	378	433	1,219
Tested for etiology, N	287	292	93	672
Positive for rotavirus, N (%)	106 (36.9)	98 (33.6)	17 (18.3)	221 (32.9)
Positive for *Salmonella*, N (%)	34 (11.8)	26 (8.9)	8 (8.6)	68 (10.1)
Positive for *Shigella*, N (%)	0	2 (0.7)	12 (12.9)	14 (2.1)
Positive for *Campylobacter*, N (%)	1 (0.3)	NT	NT	-

^a ^NTUH, National Taiwan University Hospital; MSH, Min-Shen Hospital; TCH, Buddhist Tzu-Chi General Hospital.

^b^ Number of AGE cases tested for both rotavirus and bacterial agents.

^c^ Percentage of those tested.

^d ^NT, Not tested.

### Direct medical costs for AGE

The full medical costs, including BNHI reimbursements and co-payments for children <5 years old with AGE-associated ICD9 codes based on the BNHI data are shown in Table 3. The BNHI reimbursements for AGE typically include hospital room charge and nursing fee (40.7%), pharmacy and treatment-related charges (29.3%), laboratory services (14.6%), and physician fee (15.4%) (data not shown). The values in Table 3 are full medical costs including BNHI reimbursements, co-payments, and all governmental subsidies for certain subpopulations. On average, it cost US $10.8 for each ambulatory patient visit and US $336.0 for each hospitalization due to AGE. Although children <5 years old requiring hospitalization comprised approximately 3.7% (66,260/1,770,260) of children in this age group with AGE (Table 1), over half (54.8%, 22,265,691/40,600,883) of the medical expenses were accrued by these hospitalized patients (Table 3).

**Table 3:** National health insurance reimbursements and co-pays for children <5 years old with various acute gastroenteritis ICD-9-CM codes in Taiwan in 2001.

Sectors	Medical costs (in US $) for various acute gastroenteritis associated ICD-9-CM^a^											
	
	001	002	003	004	005	007	008	009	558.9	787.91	Total	Average^b^
Outpatient	5,237	8,905	394,497	7,012	10,828	5,636	350,178	5,706,479	11,900,784	5,636	18,395,192	10.8
Inpatient	0	3,353	1,986,493	30,337	8,383	0	2,800,989	2,413,857	15,018,609	3,668	22,265,691	336.0
Total	5,237	12,259	2,380,991	37,349	19,211	5,636	3,151,167	8,120,336	26,919,393	9,304	40,660,883	-

^a ^ICD-9-CM: International Classification of Diseases, 9th revision, Clinical Modification. 002, typhoid and paratyphoid fever; 003, salmonellosis [excluding 003.2 (localized *Salmonella *infections)]; 004, shigellosis; 005, other food poisoning (bacterial); 007, other protozoan intestinal diseases; 008, intestinal infections caused by other organisms; 0086, enteritis due to specified virus; 00861, rotavirus; 009, ill-defined intestinal infections; 558.9, other and unspecified noninfectious gastroenteritis and colitis; 787.91, diarrhoea. There were no cases with the ICD-9-CM of 006 (amoebiasis) in this age group.

^b^ Total medical costs divided by total case numbers (1,704,000 for outpatients and 66,260 for inpatients) (See Table 1 for details).

### Case numbers of rotavirus AGE in children <5 years old

We used a range of 32.9–43% to estimate the total number of cases of children <5 years old hospitalized with rotavirus AGE. These numbers were based on results from our study of three hospitals, which showed that 32.9% of <5 years old children hospitalized due to AGE were attributable to rotavirus infection, as well as the study of Chen et al [9], which found 43% of the same age group of AGE patients admitted to 4 sentinel hospitals in Taiwan were due to rotavirus infection. For outpatients, we used a range of 15–35% based on the report of Glass et al [13] since the 24.0% of our three hospital data fall within this range.

The annual case number for rotavirus AGE would therefore be 255,600–596,400 (1,704,000 × 15–35%) for outpatients and 21,800–28,492 (66,260 × 32.9–43%) for inpatients, for a total of 277,400–624,892 episodes of rotavirus infections in children <5 years old needing medical care in 2001 in Taiwan (Table 4). Since the population of children <5 years old was 1,426,759 in Taiwan in 2001 [14], the incidence of rotavirus infection in children this age group would be 19.4–43.8% (277,400–624,892/1,426,759). In other words, an estimated 1 out of every 2 to 5 children <5 years old in Taiwan could have rotavirus infection that needed medical care, and the estimated hospitalization rate for rotavirus infections would be 1,528–1,997/100,000 (21,800–28,492/1,426759) in children of this age group.

**Table 4:** The direct medical cost for rotavirus acute gastroenteritis (AGE) in <5 years old children in Taiwan, 2001

Sector	Medical cost (in US $) per case	Total AGE		Estimated rotavirus AGE	
			
		Case no.	Total medical cost	Case no.	Total medical cost
Outpatient	10.8	1,704,000	18,395,192	255,600–596,400^a^	2,760,480–6,441,120
Inpatient	336.0	66,260	22,265,691	21,800–28,492^b^	7,324,800–9,573,312
Total	-	1,770,260	40,660,883	277,400–624,892	10,085,280–16,014,432

^a ^The range of 15–35% was used for estimating outpatient rotavirus AGE case number based on Glass et al., (Reference [13]).

^b ^For estimation of hospitalized rotavirus AGE case number, 32.9% was used as the lower end based on the three hospital data in the present study and 43% was used as the upper end based on study of Chen et al (Reference [9]).

The estimated medical expenses due to rotavirus infections in children <5 years old were extrapolated using the average cost per patient multiplied by the number of estimated rotavirus cases (Table 4). Based on these calculations, an estimated total medical expenditure of US $10.1–16.0 million, including US $2.8–6.4 million for outpatients and US $7.3–9.6 million for inpatients was spent in 2001 for treating rotavirus infections in young children in Taiwan.

### Comparison of medical cost for rotavirus hospitalization in Taiwan, Hong Kong, and USA

We also compared the annual direct medical costs for hospitalizations due to rotavirus infections in Taiwan (US $7–10 million) with data available from USA (US $217 million in 1996 dollar) [15] and Hong Kong (US $4 million in 2001–2003) for children <5 years old [16]. After normalization with population and gross national income (GNI) per capita for 2001 [14,17], the economic burden could be compared among different countries. Although the direct medical cost for rotavirus infections per capita was lower in Taiwan (US $0.31–0.44) than in Hong Kong (US $0.59) or USA (US $0.76), the personal economic burden indexes were similar among Taiwan (1.92–2.72), Hong Kong (2.28), and USA (2.19) when normalized for GNI per capita (Table 5).

**Table 5:** Normalized medical costs for rotavirus acute gastroenteritis (AGE) hospitalizations in Taiwan, Hong Kong, and the United States (USA)

	Taiwan	Hong Kong^b^	USA^c^
Direct medical cost for rotavirus AGE (in US $)	7–10 mil	4 mil	217 mil
Population^a^	22.9 mil	6.8 mil	285.3 mil
Medical cost per capita (A)	0.31–0.44	0.59	0.76
Gross National Incomes (GNI) per capita^a ^(B)	16,170	26,060	34,800
Normalized economic burden index due to rotavirus AGE (A/B × 10^5^)	1.92–2.72	2.28	2.19

^a ^Population and GNI per capita in 2001 was used [References 14 and 17].

^b^ The Hong Kong study was based on hospital data of 2001–2003 [reference 16].

^c  ^Because the direct medical costs estimate for USA was reported in 1996 dollars [reference 15], and we used the population and GNI data from 2001, the upper range of their sensitivity for cost estimate was used (4,346 US $ per hospitalization × 50,000 hospitalizations)

## Discussion

This is the first analysis of the prevalence and medical costs of childhood rotavirus AGE for both inpatients and outpatients in Taiwan. This analysis confirmed rotavirus as a leading cause of AGE in children <5 years old who sought medical care in Taiwan. Nearly one third (32.9%) of hospitalized AGE cases were caused by rotavirus. This finding is comparable to data reported from previous studies in other countries, which ranged from 25% to 50%. Rotavirus accounted for 30–50% of diarrhoeal hospitalization in <5 years old children in England [18], 27% in diarrhoeal consultations in El Salvador [19], and 42 % of diarrhoeal admission in Argentina [20]. In New York, rotavirus infection was reported in >30% of diarrhoea cases in children younger than 5 years of age [21].

In a separate study of 4 hospitals in Taiwan, Chen et al found that 43% of AGE cases in <5 years old children hospitalized were attributable to rotavirus [9]. There are several explanations for the difference in our data (32.9%) and that of Chen et al. In two of the hospitals we studied, over 33% of the young children with AGE hospitalized were attributable to rotavirus. The lower proportion (18%) of the patients with rotavirus AGE at the third hospital is likely due to the fact that eastern region of Taiwan where the hospital is located is the least populated mountainous area and one where bacterial dysentery remains an important infectious disease. Most other parts of Taiwan, including where the other two hospitals we studies are located, are highly urbanized and more densely populated. The lower rate of rotavirus infections in the hospital in the east might have underrepresented rotavirus in that region thus lowering our estimation for the whole country. Another possible explanation is that not all children with AGE in the three hospitals we studied had stool specimens tested for microbial etiology. Different testing practices might have led to an underestimation of the incidence of rotavirus infection. We therefore used the 43% reported by Chen et al [9] as the upper range and our 32.9% as the lower range to estimate the rotavirus disease burden in children hospitalized due to AGE in Taiwan.

The true incidence of rotavirus infections in the outpatient sectors is unknown because pediatricians tend not to perform rotavirus testing in typical cases of mild watery diarrhoea. This is especially true for the outpatient sector in Taiwan as evidenced by the fact that less than 3% of the outpatients with AGE were tested for etiology in the three hospitals we studied. We feel that the use of 15–35% range reported by Glass et al [13] provides a good estimate for outpatient rotavirus disease burden in Taiwan since rotavirus was detected in 24% of the outpatients tested in these three hospitals.

Our results indicated that the disease burden of rotavirus infections could be higher in Taiwan than many other countries. An estimated one out of every 2 to 5 children younger than 5 years of age sought medical care due to rotavirus infections in Taiwan in 2001. The risks of children in the same age group requiring an outpatient visit due to rotavirus infection were estimated to be 1 out of 7 in the United States and 1 out of 5 globally [13]. Our estimation of the annual incidence of hospitalization due to rotavirus infections of 1,528–1,997/100,000 in the <5 years old children in Taiwan is also higher than many countries, including Finland (610/100,000 in 1985–1995) [22], and Australia (750–870/100,000 in 1991–1996) [23][24]. We do not have any biological explanation for the high hospitalization rate due to rotavirus infections in Taiwan. An earlier report revealed the incidence of rotavirus disease is similar in children in different countries, regardless of the degree of development [1]. The high hospitalization rate in Taiwan might be due to the easy accessibility to and relatively low cost of health care. With the support of the National Health Insurance system, the medical costs are affordable to most of the population. Children with moderate to severe diarrhoea with early signs of dehydration are frequently admitted to the hospitals in Taiwan.

Of noteworthy is that although valuable information can be obtained from the extensive database maintained by the Bureau of National Health Insurance (BNHI) in Taiwan, very few AGE cases were coded as enteritis due to rotavirus (ICD9-00861) in the BNHI database. Although children with gastroenteritis who have no pathogen isolated should be correctly coded as ICD 009 (ill-defined intestinal infections), the BNHI data showed that ICD code 558.9 (other and unspecified noninfectious gastroenteritis and colitis) was often used instead. Earlier studies by Nelson et al on rotavirus disease burden also incorporated codes 558.9 and 787.91 (diarrhoea) into their analysis of disease burden of rotavirus infections [11]. There are several explanations for the non-specific ICD coding in Taiwan. Rotavirus AGE is clinically indistinguishable from AGE caused by other pathogens. Physicians in Taiwan also do not have the incentives to look for specific etiologic agents because insurance reimbursement is not higher for more specific diagnostic ICD coding. Stool testing is seldom done by practitioners or in local hospitals. Thus laboratory diagnosis and specific ICD coding should be encouraged to increase our understanding of the roles different etiologic agents play in causing diseases in Taiwan's patients.

We also showed that although the per-capita medical cost of rotavirus infection was lower in Taiwan than that of the United States and Hong Kong, the personal economic burden was similar among the three places when normalized for gross national incomes per capita. Therefore, people in countries with different degrees of development have similar economic burdens from rotavirus infections. Other than the direct medical costs, one must also take into account associated indirect costs including an out of pocket registration fee for each outpatient visit (US $1.5–3.0), extra diapers and laundry, special diets, transportation, lost of income from work, and additional child care needs resulting from children having rotavirus infections. Thus the social and economical burden of rotavirus infections could be in actuality much higher than the above-mentioned figures.

## Conclusion

Rotavirus remains a leading etiologic agent for acute gastroenteritis in children <5 years old in Taiwan. Even though mortality from rotavirus infection is no longer a significant problem due to easy access to medical care in Taiwan, the morbidity due to rotavirus is still very high. An estimated one out of every 2 to 5 children in the <5 years old group required medical care due to rotavirus infection in Taiwan in 2001 with an estimated annual medical expenditure of US $10–16 million. It is hoped that preventive measures, including introduction of safe and effective rotavirus vaccines, would decrease the human and economic burdens of rotavirus disease in Taiwan.

## Competing interests

LMH is currently a principal investigator and CYL is a co-investigator of a phase 3 rotavirus vaccine study funded by GlaxoSmithKline. Both LMH and CYL have received lecture fees and travel support from Merck and GlaxoSmithKline. Both companies have licensed rotavirus vaccines. The other authors declare they have no competing interests.

## Authors' contributions

CYLu was responsible for data collection and analysis, and writing of the manuscript. TLL was responsible for collection and analysis of the data from the Bureau of National Health Insurance (BNHI) database and participated in manuscript writing. CLH assisted in the BNHI data collection. YHF, CYW, YHH, LYC and CYLee participated in the collection and analysis of data from the three participating hospitals. LMH designed and supervised the execution of the study and manuscript writing. All authors have read and approved the final manuscript.

## Pre-publication history

The pre-publication history for this paper can be accessed here:


